# Effects of 5 Years Aerobic Exercise on Cognition in Older Adults: The Generation 100 Study: A Randomized Controlled Trial

**DOI:** 10.1007/s40279-021-01608-5

**Published:** 2021-12-08

**Authors:** Ekaterina Zotcheva, Asta Kristine Håberg, Ulrik Wisløff, Øyvind Salvesen, Geir Selbæk, Dorthe Stensvold, Linda Ernstsen

**Affiliations:** 1 Department of Public Health and Nursing, Faculty of Medicine and Health Sciences, Norwegian University of Science and Technology, Post Box 8905, 7491 Trondheim, Norway; 2 Norwegian National Advisory Unit for Functional MRI, Department of Radiology and Nuclear Medicine, St. Olav’s University Hospital, Trondheim, Norway; 3 Department of Neuromedicine and Movement Science, Faculty of Medicine and Health Sciences, Norwegian University of Science and Technology, Trondheim, Norway; 4 Department of Circulation and Medical Imaging, Faculty of Medicine and Health Sciences, Norwegian University of Science and Technology, Trondheim, Norway; 5 School of Human Movement and Nutrition Science, University of Queensland, Brisbane, QLD Australia; 6 Norwegian National Advisory Unit On Ageing and Health, Vestfold Hospital Trust, Tønsberg, Norway; 7 Department of Geriatric Medicine, Oslo University Hospital, Oslo, Norway; 8 Faculty of Medicine, University of Oslo, Oslo, Norway; 9 Department of Cardiology, St. Olav’s University Hospital, Trondheim, Norway

## Abstract

**Objective:**

The objective of this study was to investigate whether a 5-year exercise intervention and change in peak oxygen uptake ($V{\text{O}}_{{2{\text{peak}}}}$) is associated with cognitive function in older adults.

**Methods:**

Nine hundred and forty-five participants (48% women, mean age at study end 78.2 ± 2.02 years) from the Generation 100 Study were randomized 2:1:1 to a control group, moderate-intensity continuous training or high-intensity interval training twice weekly for 5 years. Peak oxygen uptake was measured using ergospirometry at baseline and after 5 years. Global cognition and mild cognitive impairment (MCI) were assessed with the Montreal Cognitive Assessment scale (MoCA) after 5 years.

**Results:**

Compared to the control group, the combined moderate-intensity continuous training plus high-intensity interval training (ExComb) group did not have significantly different cognitive scores (beta value 0.26, 95% confidence interval [CI] − 0.17, 0.69) or odds of MCI (odds ratio 0.86, 95% CI 0.66, 1.13). Men in the ExComb group had 0.80 points higher MoCA (95% CI 0.21, 1.40) and 32% lower odds of MCI compared with male controls (95% CI 0.47, 0.99), with no such findings in women. In the total sample, each 1 metabolic equivalent of task increase in $V{\text{O}}_{{2{\text{peak}}}}$ corresponded to 0.46 points higher MoCA (95% CI 0.25, 0.67) and 27% lower odds of MCI (95% CI 0.63, 0.85). Compared to $V{\text{O}}_{{2{\text{peak}}}}$ stable, participants whose $V{\text{O}}_{{2{\text{peak}}}}$ increased did not have significantly different cognitive scores (beta value 0.24, CI − 0.68, 1.15) or odds of MCI (odds ratio 0.70, 95% CI 0.36, 1.34), whereas participants whose $V{\text{O}}_{{2{\text{peak}}}}$ decreased had 0.64 points lower MoCA (95% CI − 1.15, − 0.14) and 35% higher odds of MCI (95% CI 0.98, 1.87).

**Conclusions:**

Overall, exercise was not significantly associated with cognition among older adults. However, maintaining or increasing $V{\text{O}}_{{2{\text{peak}}}}$ appeared to benefit cognition.

**Clinical Trial Registration:**

ClinicalTrials.gov NCT01666340.

**Supplementary Information:**

The online version contains supplementary material available at 10.1007/s40279-021-01608-5.

## Key Points

**Table:** 

In this randomized controlled trial in older adults, 5 years of moderate-intensity and high-intensity exercise did not show a significant effect on global cognition or likelihood of mild cognitive impairment.
The effect of exercise was sex specific, in that moderate-intensity and high-intensity exercise was significantly associated with better global cognition and lower odds of mild cognitive impairment in men, but not in women.
Increased peak oxygen uptake had beneficial and significant effects on cognition in both men and women.

## Introduction

As populations worldwide age, the prevalence of cognitive impairment and dementia is expected to increase substantially [1], with a recent Norwegian study suggesting that the prevalence of mild cognitive impairment (MCI) and dementia is even higher than predicted [2]. This will undoubtedly increase the burden on our healthcare systems, warranting the search for cost-effective strategies for delaying or preventing cognitive decline. Observational studies have repeatedly linked physical activity and exercise to better cognitive function [3], and lower dementia risk [4, 5]. However, results from randomized controlled trials are inconsistent, with some showing positive effects of exercise on cognition among older adults [6, 7], whereas others found no evidence of such an effect [8].

A main limitation of previous trials is the relatively short intervention duration (8 weeks–24 months), which may partially explain the conflicting results. Indeed, longer exercise interventions appear to have a greater impact on cognition [9]. Other factors that may impact study outcomes are exercise intensity and sex differences. Exercise at moderate-to-high intensity seems to be more beneficial for cognition than exercise at low or unspecified intensity [7]. Further, studies suggest a more pronounced effect of exercise on cognition in women [6, 9–11]. Altogether, this underscores the need for large long-lasting intervention studies with an emphasis on exercise intensity and sex differences to better understand the effect of exercise on cognition in older adults.

Higher levels of cardiorespiratory fitness (CRF), often measured as maximum or peak oxygen uptake ($V{\text{O}}_{{2{\text{peak}}}}$) [12], have repeatedly been associated with better cognitive function [13, 14]. According to the CRF hypothesis [15], increases in CRF are an important contributing factor in the association between exercise and cognition [15–17]. Indeed, higher CRF has been linked to several cognitive health determinants, such as larger volumes in the prefrontal cortex and hippocampus [18, 19] and greater cerebral blood flow [20, 21]. Thus, taking changes in CRF into account may contribute to clarify discrepancies in the existing literature on exercise and cognition.

In this study, we investigated the overall and sex-specific effect of a 5-year aerobic exercise intervention on cognitive function and whether the intervention was associated with MCI in 945 older adults. We also assessed the effect of change in $V{\text{O}}_{{2{\text{peak}}}}$ on cognitive function and its association with MCI.

## Methods

### Study Population

**Fig. 1:**
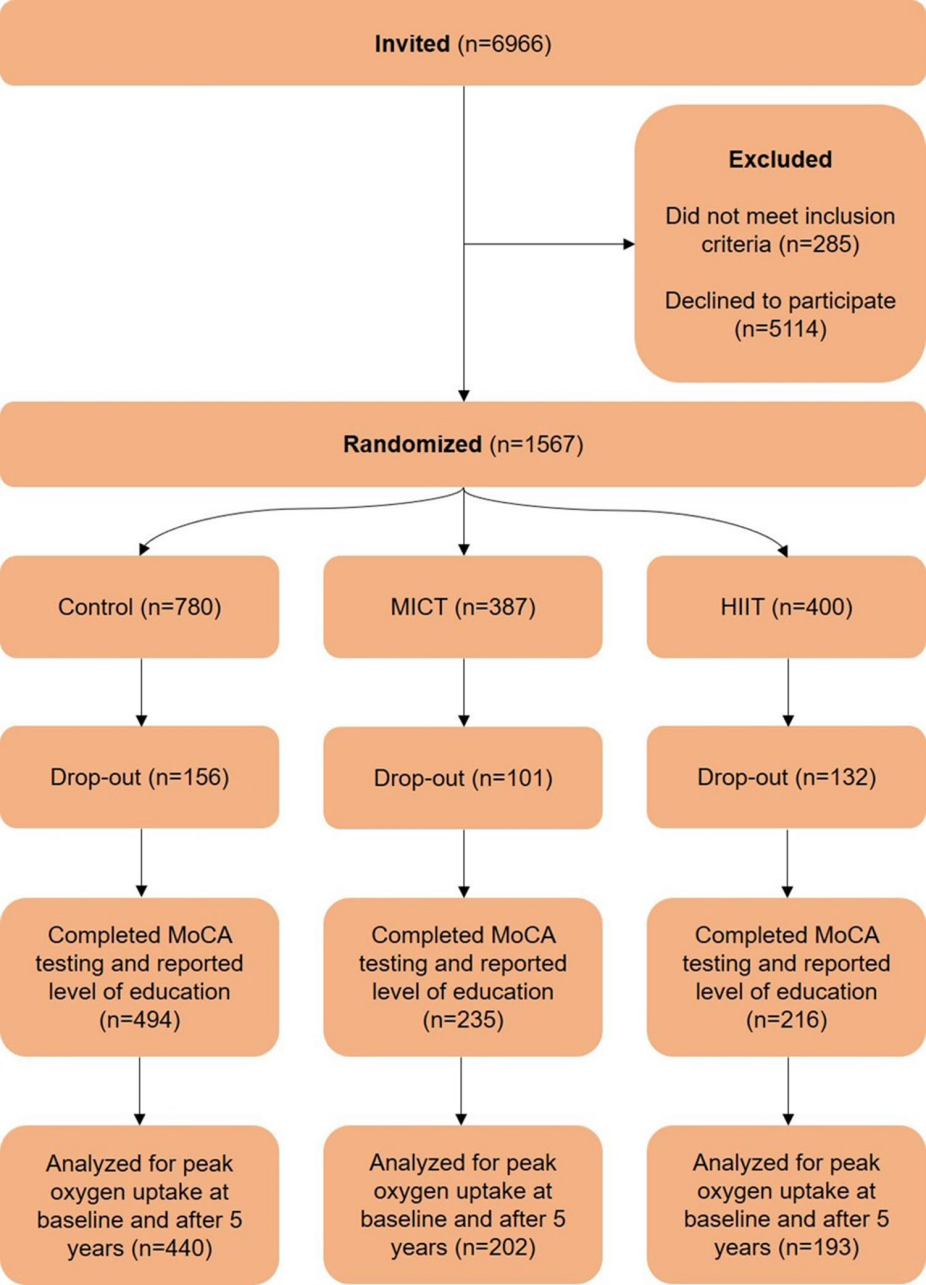
Flow chart. *HIIT* high-intensity interval training, *MICT* moderate-intensity continuous training, *MoCA* Montreal Cognitive Assessment scale

We included 945 participants with data on education and cognition at study end from the Generation 100 Study (Fig. 1). The Generation 100 Study is a randomized controlled trial designed to investigate the effect of 5 years of aerobic exercise on mortality and morbidity [22]. In 2012, all individuals born during 1936–1942 residing in the municipality of Trondheim (*n* = 6966) were invited, and 1567 individuals participated at baseline (Fig. 1). To take part in the study, participants had to be able to complete the exercise program and be free from diagnosed dementia, along with several other conditions and diseases, details described elsewhere [22].

### Design

Included participants were invited to examinations at baseline (before randomization), and after 1, 3, and 5 years. Examinations included a test of $V{\text{O}}_{{2{\text{peak}}}}$, clinical examination, and cognitive testing. Participants also filled out questionnaires regarding different aspects of health and lifestyle. For full details, see Stensvold et al. [22].

### Exercise Intervention

Participants were randomized 2:1:1, stratified by sex and cohabitation status (living with someone vs living alone), to either an unsupervised control group that was recommended to follow national recommendations for physical activity in 2012, advising 30 min of moderate-intensity physical activity almost daily [23], or to two weekly sessions of aerobic exercise over 5 years. Participants randomized to aerobic exercise performed either moderate-intensity continuous training (MICT) or high-intensity interval training (HIIT) twice weekly. Moderate-intensity continuous training consisted of 50 min of continuous aerobic exercise at moderate intensity (70% of peak heart rate), corresponding to ~ 13 on the Borg scale for ratings of perceived exertion [24], whereas HIIT comprised ~ 40 min of interval training, consisting of 4-min working periods at 85–95% of peak heart rate (~ 16 on the Borg scale) [24] with 3-min active breaks (60–70% of peak heart rate) in between. Both exercise groups were offered organized outdoor training twice weekly, consisting of activities such as walking or running. Participants could choose to attend these organized training groups or to exercise individually. In addition, participants in both exercise groups met for a supervised spinning session once every sixth week, where they wore a heart rate monitor to ensure exercise at the correct intensity [22]. Details on exercise adherence are presented in the Electronic Supplementary Material (ESM).

### Cognitive Assessment

Cognitive function was assessed using the Norwegian version [25] of the Montreal Cognitive Assessment (MoCA) [26]. The MoCA is a brief measure of global cognition designed to detect MCI, and covers cognitive domains such as short-term memory, visuospatial abilities, executive function, and orientation to time and place. Higher scores indicate better cognitive function, with a maximum possible score of 30 points [26].

The MoCA was administered to all participants directly upon arrival to the exercise laboratory. A PhD candidate or graduate student, who each went through the same standardized training and followed a standardized testing protocol, performed the assessment. Assessors were blinded to group allocation. Because of limited resources, the MoCA was not performed at baseline or at the 1-year examination. In this study, we used data from the cognitive assessment at study end, after 5 years.

We used the raw MoCA score, without adding an extra point for low educational attainment [27, 28]. As the standardized MCI cut-off of 26 has been found to produce many false positives [29, 30], we used age-specific and education-specific cut-offs from a large normative study of older Swedish individuals [31]. As Norway and Sweden are neighboring countries with similar demographics, we considered the normative data applicable in our study. MCI was defined as scoring 1.5 standard deviation (SD) below the normative mean, as recommended for detection of mild neurocognitive disorder (corresponding to MCI) in the Diagnostic and Statistical Manual of Mental Disorders, Fifth Edition [32]. Cut-offs were 21 for primary, 22 for secondary, and 24 for high educational attainment, as recommended for the age group 75–85 years [31].

### $V{\text{O}}_{{2{\text{peak}}}}$

Peak oxygen uptake (mL/kg/min) was measured using ergospirometry, details described elsewhere [33]. In our study sample, 835 participants had data on $V{\text{O}}_{{2{\text{peak}}}}$ at both baseline and at study end (see Fig. 1). Change in $V{\text{O}}_{{2{\text{peak}}}}$ (in metabolic equivalent of task [MET] values, corresponding to ~ 3.5 mL/kg/min) was assessed by subtracting baseline $V{\text{O}}_{{2{\text{peak}}}}$ from $V{\text{O}}_{{2{\text{peak}}}}$ at study end. In addition, we categorized change in $V{\text{O}}_{{2{\text{peak}}}}$ into three groups: decreased (> 1 MET decrease, *n* = 252), stable (± 1 MET change, *n* = 526), and increased (> 1 MET increase, *n* = 57).

### Baseline Data

Questionnaires were used to obtain information about sex, cohabitation status, educational attainment, smoking status, alcohol consumption in units during the past 2 weeks, history of cardiovascular disease (myocardial infarction, angina pectoris, heart failure, atrial fibrillation, other heart disease or stroke), diabetes mellitus, limiting long-term illness or injury (of physical or psychological nature, lasting a minimum of 1 year and reducing everyday functioning), 30 min of daily physical activity, sedentary time, self-reported memory problems (poor memory and remembering less than before), headaches past year, and family history of dementia. Participants completed the Hospital Anxiety and Depression Scale (HADS) [34] to assess symptoms of anxiety and depression. Waist circumference and hypertension (diastolic blood pressure ≥ 90 mmHg, systolic blood pressure ≥ 140 mmHg, or reported use of blood pressure medication) were assessed at the clinical examination.

### Statistical Analyses

We followed a predefined statistical analysis plan (ESM). The MICT and HIIT groups were combined (ExComb) and compared with the control group in the main analyses. Additionally, we investigated the association between exercise groups (MICT vs HIIT vs control) and outcome variables. Baseline characteristics are presented as numbers with percentages or means with SDs. The effect of the intervention on change in $V{\text{O}}_{{2{\text{peak}}}}$ from baseline to study end and group differences in $V{\text{O}}_{{2{\text{peak}}}}$ at baseline and study end were assessed using general linear modeling with sex, age at baseline, and cohabitation status as covariates. Beta values (B) with 95% confidence intervals (CIs) for the effect of intervention group or change in $V{\text{O}}_{{2{\text{peak}}}}$ on MoCA scores were obtained using linear regression. We also assessed quadratic relationships between changes in $V{\text{O}}_{{2{\text{peak}}}}$ and MoCA scores by fitting a linear vs quadratic regression model including squared $V{\text{O}}_{{2{\text{peak}}}}$ change and examining curve fit for the two models. Odds ratios (ORs) with 95% CIs for the association of the intervention or change in $V{\text{O}}_{{2{\text{peak}}}}$ with MCI were obtained using logistic regression. Analyses with intervention group as exposure were first performed unadjusted, and then repeated including the randomization variables sex and cohabitation status. Analyses with change in $V{\text{O}}_{{2{\text{peak}}}}$ as exposure were adjusted for sex, cohabitation status, group allocation, self-reported memory problems at baseline, and $V{\text{O}}_{{2{\text{peak}}}}$ at baseline (analyses with continuous change in $V{\text{O}}_{{2{\text{peak}}}}$ only). We decided a priori that additional analyses adjusting for possible confounders would be performed in analyses with intervention group as exposure if we observed substantial differences between intervention groups with regard to variables that could have a clinically important impact on our results.

Exploratory statistical interaction effects were examined using general linear modeling (MoCA scores) and logistic regression (MCI). As the logistic regression model used in analyses with MCI only assesses multiplicative interaction, we assessed additive interaction between dichotomous exposure variables by calculating relative excess odds due to interaction [35]. In the presence of an interaction effect, we performed stratified analyses. All analyses were performed in IBM SPSS Statistics version 26.0 (IBM Corporation, Armonk, NY, USA). A *p*-value below 0.05 was considered an indication of statistical significance.

## Results

Baseline characteristics of the study sample are presented in Table 1. Mean age of the 945 study participants at study end was 78.2 years (SD 2.02), and 47.6% were women. After 1, 3, and 5 years, respectively, adherence to prescribed exercise in our study sample was 77%, 73%, and 75% in the MICT group, and 68%, 70%, and 76% in the HIIT group. In the control group, the proportion of participants in our study sample following national guidelines for physical activity was 87%, 86%, and 96% after 1, 3, and 5 years, respectively. Details on activity patterns and sex-specific adherence are presented in Tables 2 and 3 of the ESM.

**Table 1:** Baseline characteristics by intervention group

	Control (*n* = 494)	MICT (*n* = 235)	HIIT (*n* = 216)
Mean (SD) age (years)	72.2 (2.02)	72.3 (2.05)	72.4 (2.08)
Women	239 (48.4)	114 (48.5)	97 (44.9)
Cohabitation (yes)	378 (76.5)	169 (71.9)	171 (79.2)
Higher education (college/university)	271 (54.9)	138 (58.7)	123 (56.9)
Minimum of 30 min PA daily	355 (71.9)	177 (75.3)	169 (78.2)
Mean (SD) sedentary time (hours)	5.57 (2.18)	5.96 (2.21)	5.50 (2.05)
Mean (SD) $V{\text{O}}_{{2{\text{peak}}}}$ (mL/kg/min)	29.8 (6.42)	29.8 (6.45)	30.8 (6.33)
Mean (SD) waist circumference	93.9 (10.5)	93.5 (10.9)	93.3 (10.9)
Daily smoker	16 (3.2)	9 (3.8)	5 (2.3)
Mean (SD) units of alcohol (past 2 weeks)	7.80 (7.50)	7.52 (7.86)	7.00 (7.51)
Mean (SD) depression score (HADS)	2.38 (2.11)	2.77 (2.36)	2.50 (2.05)
Mean (SD) anxiety score (HADS)	2.87 (2.43)	3.39 (2.97)	3.12 (2.40)
History of CVD	73 (14.8)	48 (20.4)	33 (15.3)
Hypertension	261 (52.8)	126 (53.6)	101 (46.8)
Diabetes mellitus	25 (5.06)	10 (4.26)	8 (3.70)
Limiting long-term illness	65 (13.2)	49 (20.9)	24 (11.1)
Self-reported memory problems	258 (52.2)	111 (47.2)	117 (54.2)
Headaches past year (yes)	52 (10.5)	34 (14.5)	18 (8.3)
Family history of dementia	65 (13.2)	37 (15.7)	39 (18.1)

*CVD* cardiovascular disease, *HADS* Hospital Anxiety and Depression Scale, *HIIT* high-intensity interval training, *MICT* moderate-intensity continuous training, *PA* physical activity, *VO*_*2peak*_ peak oxygen uptake

Values are presented as *n* (%) unless stated otherwise

### Group Differences

Mean MoCA scores and number of MCI cases by intervention group are presented in Table 2. Overall, we did not observe a significant effect of exercise on cognition. Compared to the control group, the ExComb group had higher MoCA scores (adjusted B 0.26, 95% CI − 0.17, 0.69) and lower odds of MCI (adjusted OR 0.86, 95% CI 0.66, 1.13) (Table 2). These results did not reach statistical significance. When the MICT group and HIIT group were compared separately to the control group, both groups had higher MoCA scores (MICT: adjusted B 0.21, 95% CI − 0.32, 0.73; HIIT: adjusted B 0.32, 95% CI − 0.22, 0.85) and lower odds of MCI (MICT: adjusted OR 0.92, 95% CI 0.66, 1.27; HIIT: adjusted OR 0.81, 95% CI 0.57, 1.15) (Table 2). These results were not statistically significant. No statistically significant differences were observed between the MICT and HIIT groups (Table 2).

**Table 2:** Effect of 5 years of exercise on MoCA score and association with MCI

	MoCA score			MCI		
	Mean (SD)	Unadjusted B (95% CI)	Adjusted^a^ B (95% CI)	*Number of cases* (%)	Unadjusted OR (95% CI)	Adjusted^a^ OR (95% CI)
Control	24.4 (3.44)	Ref.	Ref.	172 (34.8)	Ref.	Ref.
ExComb	24.6 (3.28)	0.25 (− 0.18, 0.68)	0.26 (− 0.17, 0.69)	143 (31.7)	0.87 (0.66, 1.14)	0.86 (0.66, 1.13)
Control	24.4 (3.44)	Ref.	Ref.	172 (34.8)	Ref.	Ref.
MICT	24.6 (3.44)	0.20 (− 0.32, 0.72)	0.21 (− 0.32, 0.73)	77 (32.8)	0.91 (0.66, 1.27)	0.92 (0.66, 1.27)
HIIT	24.7 (3.11)	0.30 (− 0.24, 0.84)	0.32 (− 0.22, 0.85)	66 (30.6)	0.82 (0.58, 1.16)	0.81 (0.57, 1.15)
MICT	24.6 (3.44)	Ref	Ref	77 (32.8)	Ref	Ref
HIIT	24.7 (3.11)	0.10 (− 0.53, 0.72)	0.11 (− 0.51, 0.73)	66 (30.6)	0.90 (0.61, 1.34)	0.89 (0.59, 1.32)

*B* unstandardized beta coefficient, *CI* confidence interval, *ExComb* combined MICT and HIIT, *HIIT* high-intensity interval training, *MCI* mild cognitive impairment, *MICT* moderate-intensity continuous training, *MoCA* Montreal Cognitive Assessment scale, *OR* odds ratio. *Ref.* reference, *SD* standard deviation

^a^Adjusted for sex and cohabitation status

### $V{\text{O}}_{{2{\text{peak}}}}$

Mean $V{\text{O}}_{{2{\text{peak}}}}$ at study end was 28.9 mL/kg/min in the ExComb group (28.4 mL/kg/min in the MICT group, 29.4 mL/kg/min in the HIIT group) and 28.1 mL/kg/min in the control group. Peak oxygen uptake was significantly higher in the ExComb group compared with the control group at study end (*p* = 0.047) but not at baseline (*p* = 0.268). At study end, the HIIT group had significantly higher $V{\text{O}}_{{2{\text{peak}}}}$ compared with the control group (*p* = 0.016) but not the MICT group (*p* = 0.171), and the MICT group did not differ significantly from the control group (*p* = 0.407). There was no significant group difference in change in $V{\text{O}}_{{2{\text{peak}}}}$ from baseline to study end (*p* = 0.152).

Each 1-MET increase in $V{\text{O}}_{{2{\text{peak}}}}$ from baseline to study end corresponded to 0.46 additional MoCA points (adjusted B 0.46, 95% CI 0.25, 0.67), and a 27% lower odds of MCI (adjusted OR 0.73, 95% CI 0.63, 0.85) (Table 3). Using a quadratic regression model of $V{\text{O}}_{{2{\text{peak}}}}$ (1-MET change) and MoCA scores did not result in a better fit than the linear model, with a negligible *r*^2^ change, and the assessment of curve fit confirmed that the relationship between $V{\text{O}}_{{2{\text{peak}}}}$ and MoCA scores is approximately linear. Participants whose $V{\text{O}}_{{2{\text{peak}}}}$ decreased > 1 MET had 0.64 points lower MoCA scores (adjusted B − 0.64, 95% CI − 1.15, − 0.14), and 35% higher odds of MCI (adjusted OR 1.35, 95% CI 0.98, 1.87) compared with the stable group, although the association with MCI did not reach statistical significance in the adjusted model (Table 3). Participants whose $V{\text{O}}_{{2{\text{peak}}}}$ increased > 1 MET had 0.24 points higher MoCA scores (adjusted B 0.24, 95% CI − 0.68, 1.15) and 30% lower odds of MCI (adjusted OR 0.70, 95% CI 0.36, 1.34), but these associations lacked precision and were not statistically significant.

**Table 3:** Effect of change in $V{\text{O}}_{{2{\text{peak}}}}$ on MoCA score and association with MCI

	MoCA score			MCI		
	Mean (SD)	Unadjusted B (95% CI)	Adjusted^a^ B (95% CI)	*Number of cases* (%)	Unadjusted OR (95% CI)	Adjusted^a^ OR (95% CI)
Change in $V{\text{O}}_{{2{\text{peak}}}}$ (1 MET)^b^	-	0.50 (0.29, 0.71)	0.46 (0.25, 0.67)	-	0.72 (0.62, 0.83)	0.73 (0.63, 0.85)
Stable	24.8 (3.19)	Ref.	Ref.	161 (30.6)	Ref.	Ref.
Decreased	24.1 (3.71)	− 0.73 (− 1.23, − 0.23)	− 0.64 (− 1.15, − 0.14)	96 (38.1)	1.40 (1.02, 1.91)	1.35 (0.98, 1.87)
Increased	25.1 (2.89)	0.24 (− 0.67, 1.16)	0.24 (− 0.68, 1.15)	13 (22.8)	0.67 (0.35, 1.28)	0.70 (0.36, 1.34)

*B* unstandardized beta coefficient, *CI* confidence interval, *MCI* mild cognitive impairment, *MET* metabolic equivalent of task, *MoCA* Montreal Cognitive Assessment scale, *OR* odds ratio, *Ref.* reference, *SD* standard deviation, *VO*_*2peak*_ peak oxygen uptake

^a^Adjusted for sex, cohabitation status, self-reported memory problems at baseline, and intervention group

^b^Additionally adjusted for VO_2peak_ at baseline

### Interaction Analyses

The effect of intervention group on MoCA score differed according to sex (interaction, *p* = 0.009). No statistically significant interaction was observed for sex and intervention group on MCI, or sex and change in $V{\text{O}}_{{2{\text{peak}}}}$, intervention group and change in $V{\text{O}}_{{2{\text{peak}}}}$, family history of dementia and intervention group, or family history of dementia and change in $V{\text{O}}_{{2{\text{peak}}}}$ on MoCA score or MCI (all *p* > 0.05). Analyses of additive interaction indicated a joint effect of sex and intervention group on MCI (adjusted relative excess odds due to interaction 0.64, 95% CI 0.05, 1.22).

Sex-specific mean MoCA scores and the number of MCI cases are presented in Table 4. Mean change in $V{\text{O}}_{{2{\text{peak}}}}$ for women was − 0.43 MET (SD 0.88) in the control group and − 0.25 MET (SD 0.98) in the ExComb group. Corresponding numbers for men were − 0.70 MET (SD 1.25) in the control group and − 0.68 MET (SD 1.20) in the ExComb group. Analyses stratified by sex revealed that men in the ExComb group had 0.80 points higher mean MoCA scores (adjusted B 0.80, 95% CI 0.21, 1.40) and 32% lower odds of MCI (adjusted OR 0.68, 95% CI 0.47, 0.99) compared with men in the control group, with no such effect in women (Table 4).

**Table 4:** Effect of 5 years of exercise on MoCA score and association with MCI, stratified by sex

	MoCA score			MCI		
	Mean (SD)	Unadjusted B (95% CI)	Adjusted^a^ B (95% CI)	*Number of cases* (%)	Unadjusted OR (95% CI)	Adjusted^a^ OR (95% CI)
Women						
Control	25.0 (3.24)	Ref.	Ref.	66 (27.6)	Ref.	Ref.
ExComb	24.6 (3.42)	− 0.35 (− 0.97, 0.27)	− 0.35 (− 0.97, 0.27)	64 (30.3)	1.14 (0.76, 1.72)	1.12 (0.74, 1.68)
Men						
Control	23.9 (3.54)	Ref.	Ref.	106 (41.6)	Ref.	Ref.
ExComb	24.7 (3.16)	0.80 (0.21, 1.40)	0.80 (0.21, 1.40)	79 (32.9)	0.69 (0.48, 1.00)	0.68 (0.47, 0.99)

*B* unstandardized beta coefficient, *CI* confidence interval, *ExComb* combined MICT and HIIT, *HIIT* high-intensity interval training, *MCI* mild cognitive impairment, *MICT* moderate-intensity continuous training, *MoCA* Montreal Cognitive Assessment scale, *OR* odds ratio, *Ref.* reference

^a^Adjusted for cohabitation status

### Additional Analyses

Separate adjustments for 30 min of daily physical activity, a family history of dementia, and a limiting long-term illness did not yield any notable changes in results (Table 1 of the ESM).

## Discussion

In the present study, 5 years of moderate-to-high intensity exercise in older adults was not significantly associated with cognition, although slightly higher cognitive scores and lower odds of MCI were observed in the ExComb group compared with the control group. Men in the ExComb group had significantly higher MoCA scores and lower odds of MCI than men in the control group, with no such effect in women, suggesting that the effect of exercise on cognition differs according to sex. Further, a change in $V{\text{O}}_{{2{\text{peak}}}}$ was significantly associated with cognition. In particular, each 1-MET increase in $V{\text{O}}_{{2{\text{peak}}}}$ was associated with higher MoCA scores and lower odds of MCI, whereas participants whose $V{\text{O}}_{{2{\text{peak}}}}$ decreased had lower MoCA scores compared with participants whose $V{\text{O}}_{{2{\text{peak}}}}$ remained stable, indicating that maintaining or improving $V{\text{O}}_{{2{\text{peak}}}}$ is important for cognitive health in older age.

### Exercise Effects on Cognition

To date, there is a lack of randomized controlled trials investigating the effect of exercise on the incidence of MCI in initially cognitively healthy older adults. In a large 24-month randomized controlled exercise trial, Sink et al. [36] found no differences in incident MCI between the exercise group and the health education group. In contrast, longitudinal observational studies have shown that moderate-to-vigorous physical activity is associated with a lower risk of incident cognitive impairment [37, 38]. This discrepancy is likely due to the need for studies to be long lasting to be able to detect incident cognitive impairment, something that is easier to achieve in observational studies. Unfortunately, given the lack of baseline assessments of MCI, we were unable to assess the incidence of new MCI cases in our study.

Although our overall results from a 5-year randomized controlled exercise trial provided limited effect sizes that did not reach statistical significance, they underscore a potential for the prevention of cognitive decline with weekly exercise at higher intensities in the general population of older adults. The population approach has been highlighted as the most crucial strategy in preventive healthcare [39], suggesting that although differences in health parameters in a study may be small at the individual level, they may play a more important role at the population-based level.

There are several plausible explanations as to why we did not observe a significant cognitive benefit of the 5-year exercise intervention. First, the assigned level and/or intensity of exercise may have been insufficient to produce a substantial group difference in global cognition or MCI in older adults, which is in line with previous findings [8, 36]. Further, it is worth noting that the control group in the Generation 100 Study had a high level of physical activity, and actually performed more HIIT exercise than the MICT group [40]. This likely influenced the lack of group differences in cognition in our study. Finally, individuals who dropped out from the study or declined to participate in the cognitive screening, and were thus not included in the analyses, may have had a different cognitive status than the included study participants. This may have led to a more cognitively homogenous sample, reducing individual and group differences in cognition.

### Sex Differences

Although the overall effect of the exercise intervention on cognition was non-significant, our results provide novel evidence suggesting that the effect of moderate-to-high intensity aerobic exercise on cognition is more pronounced in men than in women. Men in the ExComb group had 36% lower odds of MCI, which is a smaller effect than in observational studies of physical activity and cognitive impairment [37, 38], but still can be of significance to public health given that approximately 5–10% of individuals with MCI will progress to dementia annually [41]. The differences in MoCA scores between men who exercised and men in the control group are, although statistically significant, of less clinical significance [42]. Our findings are contradictory to previous studies, which found greater cognitive benefits of exercise in women than in men [6, 43]. As there was no interaction effect of sex and change in $V{\text{O}}_{{2{\text{peak}}}}$ on cognitive outcomes, and group differences in changes in $V{\text{O}}_{{2{\text{peak}}}}$ in men was minimal, differences in changes in $V{\text{O}}_{{2{\text{peak}}}}$ are unlikely to be the reason behind our findings. However, previous findings indicate higher prevalence and incidence rates of MCI in men [2, 44, 45]. Similarly, a higher proportion of men in our study were classified as having MCI at study end than women, which may have made it easier to uncover group differences in men. Additionally, several studies indicate higher resilience to age-related cognitive decline in women as opposed to men [46, 47]. As such, it is plausible that the differences in cognition observed between men in the intervention groups in our study are due to age-related cognitive decline in men in the control group, which was to some extent prevented in men in the exercise group. However, as we did not have baseline assessments of MoCA, we cannot be certain whether sex differences in the rate of age-related cognitive decline can explain the observed significant differences in men, but not in women. Thus, our conflicting results warrant further investigation of sex-dependent effects of exercise on cognition.

### $V{\text{O}}_{{2{\text{peak}}}}$

The observed association between change in $V{\text{O}}_{{2{\text{peak}}}}$ and cognition is in agreement with previous findings [13, 14, 16, 17], and with the CRF hypothesis [15]. The active control group [40] may explain why the associations between intervention group and MCI and cognitive function were marginal, whereas associations of change in $V{\text{O}}_{{2{\text{peak}}}}$ and cognition were substantial and significant, regardless of group allocation. Indeed, although $V{\text{O}}_{{2{\text{peak}}}}$ was significantly higher in the ExComb group than the control group at study end, we did not observe any significant group difference in change in $V{\text{O}}_{{2{\text{peak}}}}$ from baseline to study end. This may explain why group allocation did not have a significant effect on cognition, whereas change in $V{\text{O}}_{{2{\text{peak}}}}$ was independently associated with better cognition. Altogether, our results suggest that participating in exercise that improves or maintains CRF in older age is an effective strategy to ensure better brain health in older adults.

### Strengths and Limitations

The strengths of our study include the large sample of older adults, the long intervention duration, high adherence to the intervention, and thorough health assessments, including repeated measurements of $V{\text{O}}_{{2{\text{peak}}}}$ and detailed information on participants’ health status. The main limitation was the absence of MoCA assessments at baseline. This means that we cannot rule out the possibility of cognitively impaired participants at baseline, despite exclusion of individuals with dementia. Although a significantly uneven distribution is unlikely because of the thorough randomization process, we cannot rule out the possibility that an uneven distribution of cognitive function may have occurred at baseline. Hence, we could not assess how the exercise intervention affected change in cognitive status from baseline to study end. Another limitation was the active control group [40], which may have reduced group differences. It is difficult to obtain a suitable control group in randomized controlled exercise trials, as it is unethical to ask participants not to engage in any physical activity over a longer period. Volunteer bias [48] may have been present, as included participants were more active and more likely to report good health than non-participants [22]. Thus, the Generation 100 Study may not be representative of the general population of older adults. Adherence was assessed after 1, 3, and 5 years, and we do thus not have information on adherence between these timepoints. As adherence was measured using a questionnaire validated in young men, we cannot be sure that the questionnaire reflects the actual amount or intensity of exercise performed by the older adults in this study. Hence, we cannot rule out the possibility that some participants misinterpreted the questions, or that recent life events or cognitive changes may have caused recall bias [49]. Although the adherence criteria were set at a lower level than the prescribed exercise amount, data from the Generation 100 Study show that the participants on average exercised well above the minimum criteria [40]. Further, the power calculations in the original study were based on the primary outcome (mortality) [22] and did not take into account effect modification. The main analyses and the sex-stratified analyses were thus likely underpowered and must be interpreted with caution. Finally, we chose a different cut-off for MCI than in the original MoCA study [26], which may lower the validity of our results. However, mean MoCA scores in our study sample were below the recommended cut-off of 26 points, which supports previous findings that indicate that this cut-off is too high [29, 30].

## Conclusions

In this randomized controlled trial, 5 years of aerobic exercise in older adults was not significantly associated with global cognition and odds of MCI compared to a control group. However, stratified analyses showed that exercise was significantly and positively associated with global cognition and lower odds of MCI in men, but not women. In both men and women, increases in $V{\text{O}}_{{2{\text{peak}}}}$ were significantly associated with lower odds of MCI and higher cognitive scores, whereas decreased $V{\text{O}}_{{2{\text{peak}}}}$ was negatively associated with cognition. Our results suggest that participating in exercise that maintains or improves CRF is beneficial for cognition among older adults, but that the effects of exercise may be sex specific, warranting the assessment of sex differences in future research.

## Supplementary Information

Below is the link to the electronic supplementary material.

Supplementary file1 (PDF 112 kb)

## Declarations

### Availability of data and material

We are not permitted to share individual data from the current trial, but we are open to collaborative research with researchers worldwide, who can have access to analyzed data from our university. We have also established a biobank of blood and genetic material that we plan to share with researchers worldwide, but individual data must be analyzed within our university only.
