# Supplementary material for: Effects of 5 Years Aerobic Exercise on Cognition in Older Adults: The Generation 100 Study: A Randomized Controlled Trial
Source: Sports Med. 2021 Dec 8;52(7):1689–99. doi: 10.1007/s40279-021-01608-5 (PMC9213353; doi:10.1007/s40279-021-01608-5)
Supplement: Supplementary file 1 — Supplementary file1 (PDF 112 kb) [file 40279_2021_1608_MOESM1_ESM.pdf]

Supplementary Appendix

Effects of five years aerobic exercise on cognition in older adults - the Generation 100 Study: a randomized controlled trial.

Sports Medicine

Ekaterina Zotcheva, MSc, Asta Kristine Håberg, PhD, Ulrik Wisløff, PhD, Øyvind Salvesen, PhD, Geir Selbæk, PhD, Dorthe Stensvold, PhD, Linda Ernstsen, PhD*

*Corresponding author: Linda Ernstsen, phone no. +47 73413036, email: linda.ernstsen@ntnu.no

Supplementary Table 1. Comparison of total MoCA score and MCI between intervention groups, individually adjusted for clinically relevant variables that differed between the groups.

Covariate added to model		n	MoCA score	MCI
			Adjusted[a] B (95% CI)	Adjusted[a] OR (95% CI)
Min. 30 mins PA daily	Control	471	Ref.	Ref.
	MICT+HIIT	435	0.21 (-0.23, 0.65)	0.87 (0.67, 1.14)
	Control	471	Ref.	Ref.
	MICT	227	0.17 (-0.36, 0.71)	0.86 (0.62, 1.19)
	HIIT	208	0.25 (-0.31, 0.80)	0.89 (0.64, 1.24)
	MICT	227	Ref.	Ref.
	HIIT	208	0.07 (-0.57, 0.71)	1.04 (0.71, 1.52)
Limiting long-term illness	Control	441	Ref.	Ref.
	MICT+HIIT	406	0.11 (-0.34, 0.55)	0.87 (0.66, 1.15)
	Control	441	Ref.	Ref,
	MICT	210	0.02 (-0.52, 0.57)	0.91 (0.65, 1.27)
	HIIT	196	0.19 (-0.36, 0.75)	0.84 (0.59, 1.18)
	MICT	210	Ref.	Ref.
	HIIT	196	0.17 (-0.48, 0.82)	0.92 (0.62, 1.38)
Family history of dementia	Control	456	Ref.	Ref.
	MICT+HIIT	416	0.28 (-0.16, 0.73)	0.81 (0.62, 1.07)
	Control	456	Ref.	Ref.
	MICT	213	0.28 (-0.27, 0.82)	0.80 (0.57, 1.11)
	HIIT	203	0.29 (-0.26, 0.85)	0.84 (0.60, 1.17)
	MICT	213	Ref.	Ref.
	HIIT	203	0.02 (-0.63, 0.66)	1.05 (0.71, 1.56)

B: unstandardized beta coefficient; CI: confidence interval; HIIT: high intensity interval training; MCI: mild cognitive impairment; MICT: moderate intensity continuous training; MoCA: Montreal Cognitive Assessment scale; OR: odds ratio; PA: physical activity.

[a]Adjusted for sex, cohabitation status and the covariate listed in the corresponding row.

Adherence

Adherence to the prescribed exercise program was assessed using a validated questionnaire[1] at 1, 3, and 5 years after baseline. The questionnaire covers exercise frequency, intensity, and duration. Frequency was assessed by asking the participants to answer the question “How often do you exercise” with the response options “never” (0 days), “less than once a week” (0.5 days), “once a week” (1 day), “2-3 times per week” (2.5 days) and “almost every day” (5 days). Intensity was assessed by asking the participant to determine their mean intensity of exercise on the Borg scale for ratings of perceived exertion (6-20)[2]. Duration was assessed by asking the participants to answer the question “How long did you exercise each time?” with the response options “less than 15 minutes” (7.5 minutes), “15-29 minutes” (22.5 minutes), “30 minutes to 1 hour” (45 minutes), and “more than 1 hour” (60 minutes).

Adherence in the MICT group was defined as at least 30 minutes of weekly exercise corresponding to 11-14 on the Borg scale. In the HIIT group, the requirements for adherence were at least 30 minutes weekly of exercise at ≥15 on the Borg scale. Requirements for adherence in the control group were ≥75 minutes a week of physical activity.

Supplementary Table 2. Intervention adherence for study sample (n=945) by intervention group after 1, 3, and 5 years.

	1 year	3 years	5 years
**Control**			
PA recommendations	**87%**	**86%**	**96%**
MICT	55%	54%	55%
HIIT	28%	29%	27%
**MICT**			
PA recommendations	83%	82%	80%
MICT	**77%**	**73%**	**75%**
HIIT	14%	19%	17%
**HIIT**			
PA recommendations	88%	84%	79%
MICT	26%	25%	15%
HIIT	**68%**	**70%**	**76%**

HIIT: high intensity interval training; MICT: moderate intensity continuous training; PA: physical activity.
Bold numbers indicate adherence to prescribed program.

Supplementary Table 3. Intervention adherence for study sample (n=945) by intervention group and sex after 1, 3, and 5 years.

	1 year		3 years		5 years	
	Women	Men	Women	Men	Women	Men
**Control**						
PA recommendations	**90%**	**84%**	**88%**	**84%**	**98%**	**95%**
MICT	62%	48%	59%	49%	58%	52%
HIIT	24%	31%	23%	34%	23%	29%
**MICT**						
PA recommendations	86%	80%	87%	78%	83%	78%
MICT	**83%**	**70%**	**84%**	**62%**	**84%**	**66%**
HIIT	7%	20%	8%	30%	8%	25%
**HIIT**						
PA recommendations	87%	88%	89%	81%	81%	77%
MICT	32%	21%	29%	21%	20%	12%
HIIT	**65%**	**71%**	**69%**	**71%**	**76%**	**75%**

HIIT: high intensity interval training; MICT: moderate intensity continuous training; PA: physical activity.
Bold numbers indicate adherence to prescribed program.

References

1. Kurtze N, Rangul V, Hustvedt BE, Flanders WD. Reliability and validity of self-reported physical activity in the Nord-Trondelag Health Study (HUNT 2). Eur J Epidemiol. 2007;22(6):379-87. doi: 10.1007/s10654-007-9110-9.
2. Borg GA. Psychophysical bases of perceived exertion. Med Sci Sports Exerc. 1982;14(5):377-81.
